# Stability analysis of loess fill slope supported by frame prestressed anchors considering tensile strength cut-off

**DOI:** 10.1038/s41598-024-53692-y

**Published:** 2024-02-06

**Authors:** Zhuangfu Zhao, Yanpeng Zhu, Hongjie Hou, Lian Li

**Affiliations:** 1 Chengdu Technological University, Yibin, 644000 China; 2 School of Civil Engineering, Lanzhou University of Technology, Lanzhou, 730050 China

**Keywords:** Engineering, Civil engineering

## Abstract

In the stability analysis of loess fill slope, the fissure nature of loess is often ignored, which makes the stability calculation of fill slope too conservative. Based on the upper limit theory of plastic limit analysis, the stability analysis model of loess-filled fissured slope supported by frame prestressed anchors was established. Considering the tensile strength cut-off yield property of soil, the stability coefficient of slope was calculated, and the influence of different factors on slope stability was analyzed. The results show that ignoring the fissures in loess will overestimate the stability of the fill slope, and the support structure can significantly improve the stability of the loess-filled fissure slope. The research results of this paper can further enrich the stability analysis theory of loess-filled fissured slope supported by frame prestressed anchors, which is of great significance to guide engineering practice.

## Introduction

**Figure 1:**
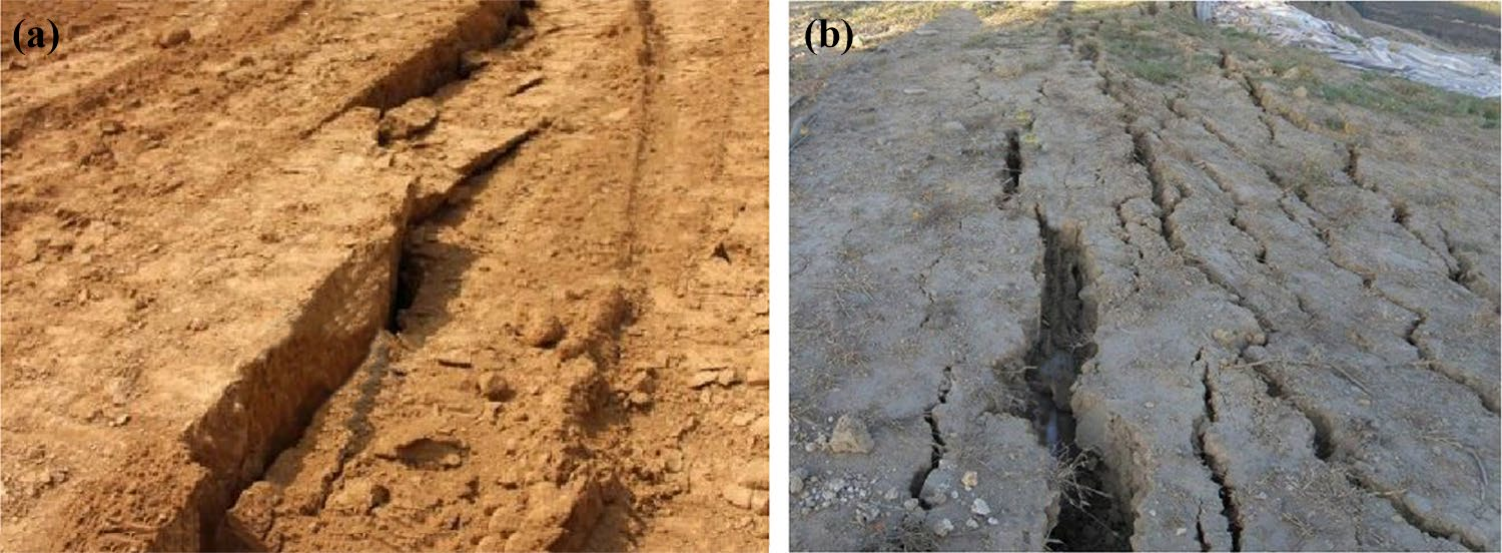
Loess fill fissures (**a**) Uneven settlement fissures in the fill area (**b**) Fissures at the top of the slope of the fill.

With the rapid economic development of northwest China, a large number of projects of "Cutting Mountains, Filling Ditches, And Creating Land" have been carried out to alleviate the scarcity of land resources, accompanied by many high-fill foundations and high-fill slope projects with loess as filler^1,2^. Due to the difference of filling quality and filling thickness, consolidation settlement, collapsible settlement and uneven settlement are easy to occur under the action of self-weight load and other external factors (e.g., earthquake)^3,4^. The macroscopic performance is that the slope produces fissures (Fig. 1). In order to avoid geological disasters of loess high fill, some engineering measures are needed. With the development of slope anchoring technology, some flexible support structures are gradually applied to slope engineering, which have achieved good support effects^5–8^. However, the cooperative working principle of retaining structure and fill is complicated, especially in the analysis theory of anchorage of fill slope, there are still many problems.

Unlike rocks, the tensile strength of loess is very low, and it is generally believed that as soon as tensile stress occurs, the soil body will undergo tensile damage producing cracks^9^. Cracks are common in loess slopes, and studies have shown that the presence of cracks can greatly reduce the stability of slopes and increase the possibility of their destabilization damage^10,11^. Therefore, it is urgent and necessary to study the effect of cracks on loess fill slopes.

Some scholars have studied fissured slopes, Zhang and Yang^10^ studied the seismic stability of slopes considering tensor fissures using a proposed dynamic method. Utili^12^ developed an assessment of slope stability considering the depth and location of fissures and obtained a series of design charts. Michalowski^13,14^ considered the formation of fissures as part of the slope damage mechanism and calculated the fissure formation. The dissipated energy during the process was calculated, and it was argued that ignoring the formation of fissures would overestimate the stability of slopes. Xu et al.^15^ derived elastic solutions for fissure depths and showed that the stability of slopes would be significantly affected at larger fissure depths. Li et al.^16^ combined the limit analysis upper limit method to derive the equation for calculating the safety factor of fissured slopes under seismic effects and analyzed the effects of different parameters on slope stability under nonlinear conditions. Unfortunately, according to the existing research results, most of them are for the unsupported fissured slope, and there are few studies on supported fissured slopes. In particular, there are few reports on the research of frame prestressed anchor supporting loess fill fissured slope.

In this paper, the tensile strength cut-off yield property of soil is used to characterize the fissure properties of loess-filled slopes. Based on the upper limit theory of plastic limit analysis, the stability analysis model of loess-filled fissured slope supported by frame prestressed anchors is established. Considering fissure characteristics, dry fissure and water-filled fissure, the general solution of stability safety factor of loess-filled slope is derived, and the stability between the supported and the unsupported is compared and analyzed by engineering examples. The research results of this paper can further enrich the theory of stability analysis of loess fissured slope supported by frame prestressed anchors, which is of great significance to guide engineering practice.

## Stability calculation of loess fissured slopes

### Fissure characterization of loess fill slope

**Figure 2:**
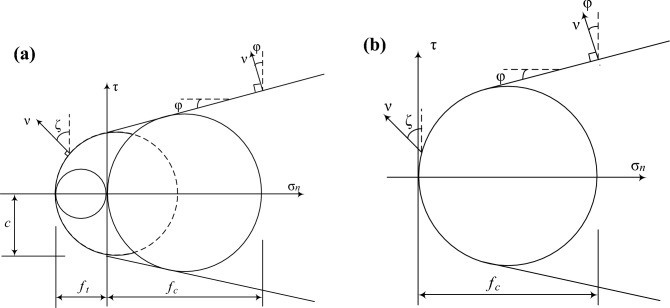
Yield conditions: (**a**) with tensile strength, (**b**) tensile strength cut-off.

In loess-filled slopes, fissures are common, research shows that the existence of fissures will greatly reduce the stability of slopes and increase the possibility of destabilization damage^11^. For materials with tensile strength, the yield properties of the soil are shown by the bending envelope including the uniaxial tensile strength $f_{t}$ in the range where fissures are expected to form, as shown in Fig. 2a, and the yield properties in other regions of the stress state are characterized by the classical Mohr–Coulomb function.

In the limit analysis, using the normal flow law, the volumetric strain rate can be defined by the shear expansion angle $\psi$ for the nonlinear part of the yield condition, $\psi { = }\zeta$ and $\psi { = }\varphi$ for the other parts, where $\zeta$ is the angle formed by the velocity discontinuity vector $v$ with the fissure opening direction and $\varphi$ is the friction angle within the soil. The normal flow law requires the velocity discontinuity vector $v$ to be perpendicular to the yield envelope, and the analytical solution of the power dissipation rate is obtained from the geometric relationship in Fig. 2a ^17^:

1 $$d_{c} = v\left( {f_{c} \frac{1 - \sin \zeta }{2} + f_{t} \frac{\sin \zeta - \sin \varphi }{{1 - \sin \varphi }}} \right)$$

where $f_{c}$ is the one-dimensional compressive strength.

For materials that do not have tensile strength, i.e. tensile strength cut-off, the yield characteristics of the soil are shown in Fig. 2b. Since the tensile strength of loess fill is generally low and considered as no tensile strength when it contains fissures, the tensile strength cut-off property of soil can be used to characterize the fissures of loess fill slope. For the material with tensile strength truncation, $f_{t} = 0$, and $f_{c} { = }2c\cos \varphi /(1 - \sin \varphi )$^17^, where $c$ is the cohesive forces. Substituting them into Eq. (1), we can obtain^14^:

2 $$d_{c} = cv\cos \varphi \frac{1 - \sin \zeta }{{1 - \sin \varphi }}$$

For slopes with existing fissures, the work consumed by the fissure formation process is no longer considered and is taken from $d_{c} = 0$ .

### Calculation assumption

**Figure 3:**
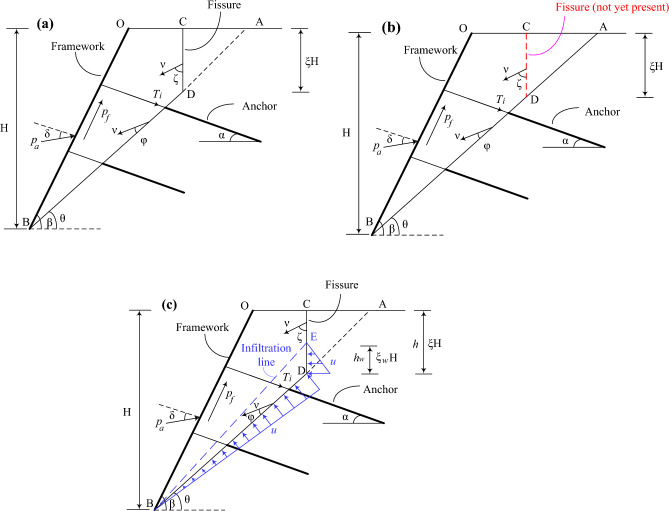
Linear failure mode: (**a**) pre-existing fissures (**b**) Considering the fissure characteristics of loess (c) pre-existing fissures and filled with water.

The common frame supporting structure includes frame anchor structure and frame anchor plate structure^18^. According to the different fissure cases, the damage mode of loess-filled fissured slopes supported by frame prestressed anchors can be divided into three different cases, take the linear damage mode as an example, as shown in Fig. 3a The slope has pre-existing fissures (i.e. dry fissures). (b) Considering the fissure nature of loess, i.e. considering the soil tensile strength cut-off property. It is considered that fissures do not exist before slope failure, and the formation of fissures is regarded as a part of slope failure mechanism, which requires energy consumption. (c) The slope has pre-exists fissures and the fissures contain water at a certain depth (i.e. water-filled fissures). It is important to note that the geometry of the slope damage mechanism may pass through or under the foot of the slope, what discussed in this paper are only slopes with a horizontal surface at the top of the slope and with the damage mechanism passing through the foot of the slope, and the insertion length of the anchor is deep enough to pass through the sliding surface of the slope.

For calculation purposes, the following assumptions were made^12–14,19^:

(1) The analysis is carried out by plane strain problem, considering that the slope surface is supported by a support structure, so the fissure is only located at the top of the slope, and the slider *OBDC* is defined by the shear slip surface *BD* as well as the existing fissure or pre-cracked surface *CD.*

(2) Slip-soil bodies are ideally rigid-plastic, obeying the linear Mohr–Coulomb law and satisfying the associated flow law;

(3) The seismic forces are applied by the proposed static method, and the horizontal and vertical seismic forces are expressed by $k_{h} W$ and $k_{v} W$, respectively, where $W$ is the weight of the sliding soil mass. $k_{h}$ and $k_{v}$ are the horizontal and vertical seismic acceleration coefficients, respectively, and $k_{v} = \lambda k_{h}$, with $\lambda$ being the scaling factor.

(4) The shear strength parameters of geotechnical materials $c$ and $\varphi$ do not change due to seismic action.

(5) Under the condition of water in the fissure, it is assumed that the soil below the infiltration line is saturated, the shear strength parameters $c$ and $\varphi$ of the soil above the infiltration line are not affected, and the water filling the fissure does not penetrate around the soil.

### Calculation of stability factor

There are various damage mechanisms of slopes, including linear, arc and logarithmic spiral damage, etc. For the sake of simplicity of derivation, the linear damage mechanism is firstly analyzed as an example, and other damage mechanisms can be derived accordingly.

#### First case: Pre-existing fissures in the slope (dry fissures) .

Firstly, considering that the slope has pre-existing fissures, as shown in Fig. 3a, the parameter $\xi$ is introduced to represent the ratio of fissure depth $h$ to slope height $H$: $\xi { = }h/H$. According to the geometric relationship, $0 \le \xi \le 1 - \tan \theta /\tan \beta$ is derived. $\beta$ is the angle between the slope and the horizontal plane, and $\theta$ is the angle between the slip surface and the horizontal plane. According to Reference^20^, it can be taken as: $\theta = (\beta + \varphi )/2$. For the loess fill slope supported by frame prestressed anchors, the external force power $\dot{W}_{OUT}$ includes the soil gravity power $\dot{W}_{G}$, horizontal seismic force power $\dot{W}_{EH}$ and vertical seismic force power $\dot{W}_{EV}$. The specific expressions are given as below.

The external power is:

3 $$\dot{W}_{OUT} = \dot{W}_{G} + \dot{W}_{EV} + \dot{W}_{EH}$$

where the soil gravity power is the external power exerted by the soil gravity of the slider *OBDC*:

4 $$\dot{W}_{G} = v\sin \left( {\theta - \varphi )\frac{{\gamma H^{2} }}{2}} \right)\left[ {\left( {1 - \xi^{2} } \right)\cot \theta - \cot \beta } \right]$$

where $\gamma$ is the weight of the soil.

The horizontal seismic force power is the external power of the slider *OBDC* under the action of the horizontal seismic inertia force, which can be presented as follows:

5 $$\dot{W}_{EH} = k_{h} v\cos \left( {(\theta - \varphi )\frac{{\gamma H^{2} }}{2}} \right)\left[ {1 - \xi^{2} \cot \theta - \cot \beta } \right]$$

The vertical seismic force power is the external power of the slider *OBDC* under the action of the vertical seismic inertia force, which can be presented as follows:

6 $$\dot{W}_{EV} = k_{v} v\sin \left( {(\theta - \varphi )\frac{{\gamma H^{2} }}{2}} \right)\left[ {\left( {1 - \xi^{2} } \right)\cot \theta - \cot \beta } \right]$$

The internal energy dissipation power $\dot{D}_{IN}$ includes the energy consumption of the slip surface *BD*: $\dot{D}_{BD}$, the power made by the anchor resistance *T*: $\dot{D}_{T}$ , the power exerted by the active earth pressure resistance *P*_*a*_: $\dot{D}_{A}$, and the power exerted by the wall-soil adhesion force *P*_*f*_: $\dot{D}_{F}$ . The expression for the power dissipated by internal energy can be presented as follows:

7 $$\dot{D}_{IN} = \dot{D}_{BD} + \dot{D}_{T} + \dot{D}_{A} + \dot{D}_{F}$$

where the energy consumption of the sliding surface *BD* can be expressed as follows:

8 $$\dot{D}_{BD} = cl_{BC} v\cos \varphi = cv\cos \varphi \frac{H}{\sin \theta }(1 - \xi )$$

where $l_{BC}$ is the length of the slip plane *BC*: $l_{BC} { = }H(1 - \xi )/\sin \theta$ .

For the frame prestressed anchor structure, the earth pressure on the retaining wall is transmitted through the frame to the anchor rod, which is balanced by the anchor rod resistance, and the power exerted by the anchor rod pullout resistance can be expressed as follows:

9 $$\dot{D}_{T} = \sum\limits_{i = 1}^{n} {T_{i} v\cos (\alpha + \theta - \varphi )}$$

where $n$ is the number of rows of anchor rods and $T_{i}$ is the resistance provided by the *i*_*th*_ anchor rod: $T_{i} = \pi D_{i} q_{sik} L_{ai}$. $D_{i}$ and $L_{ai}$ are the diameter of the anchor and the effective anchorage length of the *i*_*th*_ anchor rod, respectively. $L_{ai}$ approximates the length of the anchorage section of the anchor rod $l_{ai}$, and $q_{sik}$ is the standard value of the ultimate frictional resistance of the soil.

The frame structure provides resistance to balance the earth pressure, where the power exerted by the active earth pressure resistance provided by the retaining plate can be expressed as:

10 $$\dot{D}_{A} = P_{a} v\sin (\delta + \varphi + \beta - \theta )$$

Recommended by Technical Specification for Construction Slope Engineering (GB50330-2013)^21^, the model for calculating soil pressure of anchor retaining wall is applied, and the total active soil pressure is shown as: $P_{a} = \gamma H^{2} K_{a} /2$ . The earth pressure action position is *H/3* from the foot of the slope. $K_{{\text{a}}}$ is the Coulomb active earth pressure coefficient, which can be presented as follows:

11 $$K_{{\text{a}}} { = }\frac{{\cos^{2} \left( {\varphi_{D} - \beta + \frac{\pi }{2}} \right)}}{{\cos^{2} \left( {\beta - \frac{\pi }{2}} \right)\cos \left( {\beta - \frac{\pi }{2} + \delta } \right)\left[ {1 + \sqrt {\frac{{\sin \left( {\varphi_{D} + \delta } \right)\sin \varphi_{D} }}{{\cos \left( {\beta - \frac{\pi }{2} + \delta } \right)\cos \left( {\beta - \frac{\pi }{2}} \right)}}} } \right]^{2} }}$$

$\delta$ is the friction angle between the soil and the retaining wall surface, which can be expressed as: $\delta { = }0.7\varphi_{D}$^21^, where $\varphi_{D} = \arctan [\tan \varphi + {\text{c/(}}\gamma H)]$.

In addition, the resistance provided by the frame structure also includes wall-soil adhesion forces, according to the literature^22^, the wall-soil adhesion force can be expressed as:

12 $$p_{f} = \frac{{cl_{OB} \tan \delta }}{\tan \varphi } = \frac{cH\tan \delta \cot \varphi }{{\sin \beta }}$$

The power made by the wall-soil adhesion $\dot{D}_{F}$ can be expressed as:

13 $$\dot{D}_{F} = \frac{cH\tan \delta \cot \varphi }{{\sin \beta }}v\cos (\beta + \varphi - \theta )$$

The ratio of internal energy dissipation power and external power is taken as the overall stability coefficient of the slope^19,20^, and the overall stability coefficient of the high-fill loess slope with frame prestressed anchors based on the upper limit theorem is obtained as:

14 $$F_{s} = \frac{{\dot{D}_{IN} }}{{\dot{W}_{OUT} }} = \frac{{\dot{D}_{BD} + \dot{D}_{T} + \dot{D}_{A} + \dot{D}_{F} }}{{\dot{W}_{G} + \dot{W}_{EV} + \dot{W}_{EH} }}$$

#### Second case: Considering the fissure characteristics of loess

In this case, it is considered that there is no fissures before slope damage, and the formation of fissure is considered as part of the damage mechanism, as shown in Fig. 3b. Fissure formation requires dissipation of certain energy, and according to Eq. (2), the energy consumption of generating a fissure *CD* can be expressed as:

15 $$\dot{D}_{CD} = cv\cos \varphi \frac{1 - \sin \zeta }{{1 - \sin \varphi }}\xi H$$

According to the reference^13^, $\zeta$ can take the following values:

16 $$\zeta = \pi /2 - \theta - \varphi$$

Thus, the expression of Eq. (15) can be written as:

17 $$\dot{D}_{CD} = cv\cos \varphi \frac{1 - \cos (\theta - \varphi )}{{1 - \sin \varphi }}\xi H$$

At this time, the external power can be expressed as: $\dot{W}_{OUT} = \dot{W}_{G} + \dot{W}_{EV} + \dot{W}_{EH}$ and the internal energy dissipation power is: $\dot{D}_{IN} = \dot{D}_{BD} + \dot{D}_{CD} + \dot{D}_{T} + \dot{D}_{A} + \dot{D}_{F}$. The overall stability coefficient of the slope is

18 $$F_{s} = \frac{{\dot{D}_{IN} }}{{\dot{W}_{OUT} }} = \frac{{\dot{D}_{BD} + \dot{D}_{CD} + \dot{D}_{T} + \dot{D}_{A} + \dot{D}_{F} }}{{\dot{W}_{G} + \dot{W}_{EV} + \dot{W}_{EH} }}$$

#### Third case: Pre-existence of fissures and water in the fissures (water-filled fissure)

In the third case, considering that the fissure is pre-existing and contains water at a certain depth, as shown in Fig. 3c. It should be noted that according to the assumption (5), the water filling the fissure does not penetrate around the soil. The parameter $\xi_{w}$ is introduced to represent the ratio of the water depth in the fissure $h_{w}$ to the slope height $H$, and the fissure contains water at a depth $\xi_{w} H$ . It is assumed that water, entering the fissure, infiltrates along the slope and exits from point *B* at the foot of the slope. According to Reference^23^, the infiltration line is simplified to a straight line segment between the highest point *E* of water level depth in the fissure and the point *B* at the foot of the slope.

At this point, the gravitational power of the slider *OBDC* is divided into two parts, according to the assumption (5), the slider *OBEC* above the infiltration line takes the natural weight of the soil $\gamma$, and the slider *BDE* below the infiltration line takes the saturated weight of the soil $\gamma_{sat}$. The gravitational power of the slider *OBDC* is obtained as:

19 $$\dot{W}_{G} ^{\prime} = v\sin (\theta - \varphi )\left\{ \begin{gathered} \frac{{\gamma H^{2} }}{2}[(1 - \xi^{2} - \xi_{w} + \xi \xi_{w} )\cot \theta - \cot \beta ] \hfill \\ + \frac{{\gamma_{sat} H^{2} }}{2}(\xi_{w} - \xi \xi_{w} )\cot \theta \hfill \\ \end{gathered} \right\}$$

Horizontal seismic force power $\dot{W}_{EH} ^{\prime}$ :

20 $$\dot{W}_{EH}^{\prime } = k_{h} v\sin (\theta - \varphi )\left\{ \begin{gathered} \frac{{\gamma H^{2} }}{2}\left[ {(1 - \xi^{2} - \xi_{w} + \xi \xi_{w} )\cot \theta - \cot \beta } \right] \hfill \\ + \frac{{\gamma_{sat} H^{2} }}{2}(\xi_{w} - \xi \xi_{w} )\cot \theta \hfill \\ \end{gathered} \right\}$$

Vertical seismic force power $\dot{W}_{EV} ^{\prime}$ :

21 $$\dot{W}_{EV} ^{\prime} = k_{v} v\sin (\theta - \varphi )\left\{ \begin{gathered} \frac{{\gamma H^{2} }}{2}[(1 - \xi^{2} - \xi_{w} + \xi \xi_{w} )\cot \theta - \cot \beta ] \hfill \\ + \frac{{\gamma_{sat} H^{2} }}{2}(\xi_{w} - \xi \xi_{w} )\cot \theta \hfill \\ \end{gathered} \right\}$$

When the water in the fissure reaches a certain height, hydrostatic pressure will be generated along the fissure as well as the slip surface. The combined water pressure within the range of the fissure filling height *EC*, which can be expressed as: $u_{1} = \gamma_{w} H^{2} \xi_{w}^{2} /2$. The combined water pressure formed on the slip surface *BC* is: $u_{2} = \gamma_{w} H^{2} \xi_{w} \left( {1 - \xi } \right)/2\sin \theta$, where $\gamma_{w}$ is the unit weight of water, from which the external power of the water pressure is obtained as:

22 $$\dot{W}_{W} = \frac{v}{2}\gamma_{w} H^{2} \xi_{w}^{2} \sin \zeta + \frac{{\gamma_{w} H^{2} \xi_{w} \left( {1 - \xi } \right)}}{2\sin \theta }v\sin \varphi$$

$\eta$ is introduced to indicate the degree of water filling in the fissure: $\eta = \xi_{w} /\xi$, in the range of 0–1. When $\eta = 0$ , there is no water pressure, when $\eta = 1$ , the fissure is filled with water. At this time, the external power is: $\dot{W}_{OUT} = \dot{W}_{G} ^{\prime} + \dot{W}_{EV} ^{\prime} + \dot{W}_{EH} ^{\prime} + \dot{W}_{W}$, and the internal energy dissipation power is:$\dot{D}_{IN} = \dot{D}_{BD} + \dot{D}_{T} + \dot{D}_{A} + \dot{D}_{F}$. The slope stability coefficient is obtained as follows:

23 $$F_{s} = \frac{{\dot{D}_{IN} }}{{\dot{W}_{OUT} }} = \frac{{\dot{D}_{BD} + \dot{D}_{T} + \dot{D}_{A} + \dot{D}_{F} }}{{\dot{W}_{G} ^{\prime} + \dot{W}_{EV} ^{\prime} + \dot{W}_{EH} ^{\prime} + \dot{W}_{W} }}$$

$F_{s}$ is a function of the unknowns $\xi$ and $\theta$: $f(\xi ,\theta )$. The process of finding the minimum safety coefficient is an optimality search process for the unknowns $\xi$ and $\theta$, and the objective function is the minimum stability coefficient by genetic algorithm (*GA*) search. $\xi$ and $\theta$ are the variables of the objective function, and the stability coefficient $F_{s}$ needs to satisfy the following equation:

24 $$\frac{{\partial F_{s} }}{\partial \xi } = 0,\;\frac{{\partial F_{s} }}{\partial \theta } = 0$$

Then the minimum stability factor is:

25 $$\min F_{s} { = }f(\xi ,\theta )$$

#### Logarithmic spiral failure mechanism

**Figure 4:**
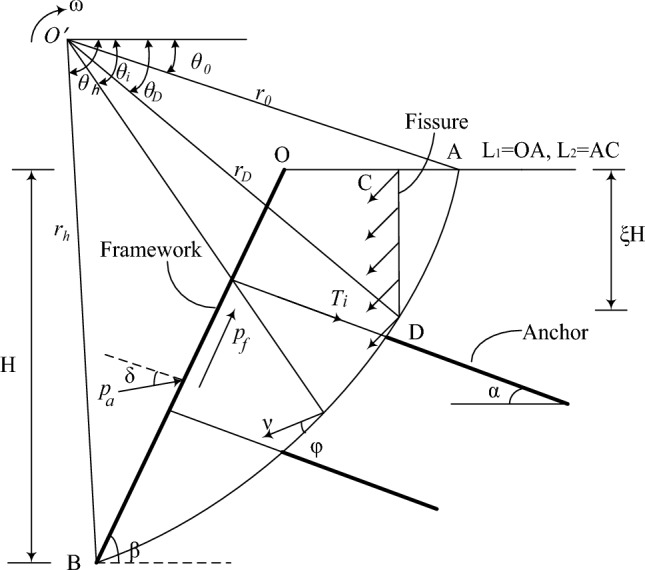
logarithmic spiral failure mode.

It should be noted that the linear failure is a relatively simple failure form for the stability analysis of uniform slopes, and the case in Fig. 3 is only used to illustrate this method. In fact, arc failure or logarithmic spiral rotation mechanism is more widely used at present. The logarithmic spiral failure mechanism is shown in Fig. 4. The failure surface is a logarithmic spiral function, and the sliding block *OBDC* rotates around the point *O’* with the angular velocity *ω*. The fissure is generated and developed along the *CD*. The solution of logarithmic spiral failure mechanism is more complex than that of linear failure, but their calculation ideas are consistent. Due to space limitation, they are not described here.

It is worth noting that the calculation method of fissure *CD* energy consumption is different from that of linear failure, because the size of the discontinuous vector and its dip angle along the fissure *CD* are not constant, but change along the *CD*. The specific solution can be seen in Reference^14^, which is expressed as follows:

26 $$\dot{D}_{CD} = r_{0}^{2} \omega \int\limits_{{\theta_{C} }}^{{\theta_{D} }} {\left( {\frac{{\sin \theta_{0} }}{{\tan \theta_{0} }}\frac{1}{\cos \theta }} \right)}^{2} \left( {f_{c} \frac{1 - \sin \theta }{2} + f_{t} \frac{\sin \theta - \sin \varphi }{{1 - \sin \varphi }}} \right)\frac{d\theta }{{\cos \theta }}$$

## Example analysis

In this section, an example is used to verify the method in this paper, and the stability of the loess fill fissured slope supported by frame prestressed anchor is analyzed. A loess fill project has a slope height of 12 m and a slope angle of 80 degrees. The slope soil parameters are shown in Table 1. The slope is supported by frame prestressed anchors, and the design results of anchors are shown in Table 2. The number of rows of anchors is: $n = 5$ (from top to bottom, the sequence numbers 1–5), and the angle of bolts is: $\alpha = 20^{ \circ }$.

**Table 1:** Soil parameters.

$\gamma$(kN/m^3^)	$c$(kPa)	$\varphi$(°)	$q_{sik}$ kN/m^2^)
19	35	20	50

**Table 2:** Design result of anchors.

Sequence number	Diameter of anchor $D$ (m)	Length of free section of anchor $l_{f}$ (m)	Length of anchorage section $l_{a}$ (m)	Total length of anchor $l$ (m)
1	150	6	10	16
2	150	5	9	14
3	150	4	8	12
4	150	3	7	10
5	150	3	5	8

### Verification of the method in this paper

**Figure 5:**
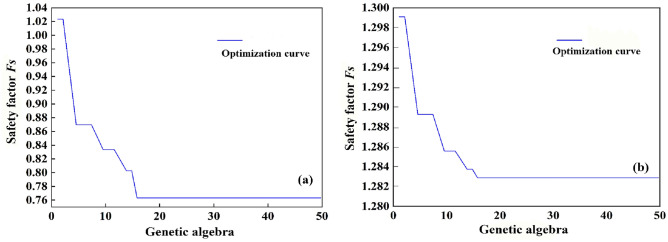
Optimization curve of safety factor (**a**) unsupported (**b**) supported.

Firstly, the feasibility of the calculation method in this paper is verified, and the safety factors of the unsupported slope and the slope supported by frame prestressed anchor are solved by genetic algorithm optimization respectively when there is no fissure (that is, the complete slope). The horizontal seismic coefficient are: $k_{h}$ = 0.2, $\lambda { = }1$. Figure 5 shows the optimization process of genetic algorithm, in which the minimum safety coefficient result is 0.76 for unsupported slope and 1.283 for supported slope, with the safety coefficient improved by about 68%. It can be seen that the stability of supported slopes has been greatly improved.

**Figure 6:**
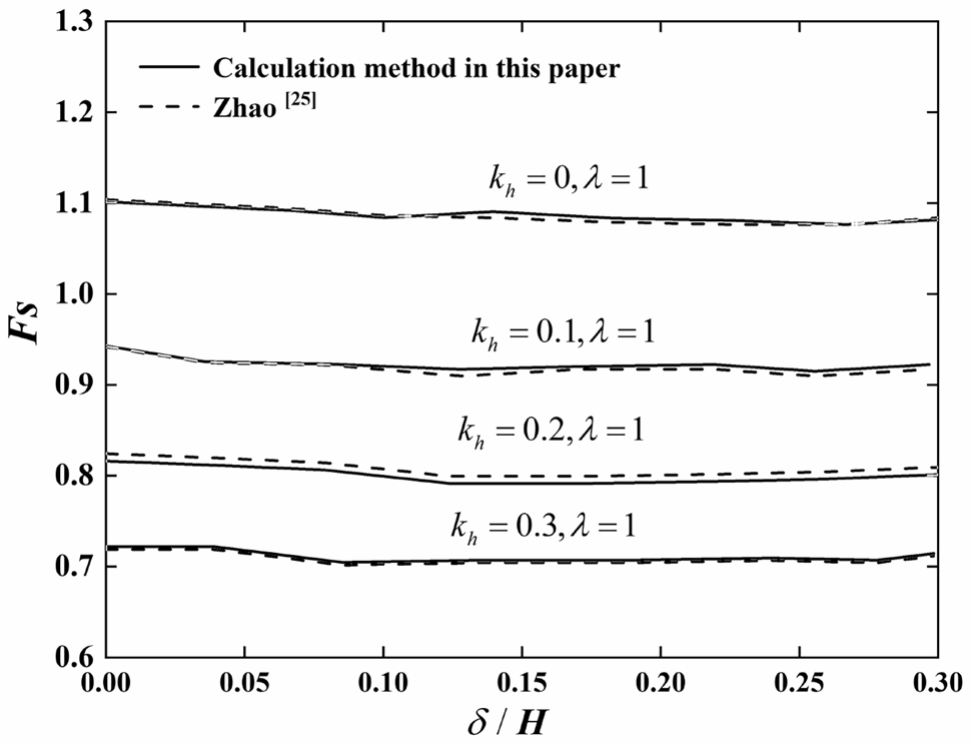
Comparative analysis of data.

In order to verify the reliability of the calculated data in this paper, through the above cases, the research results are compared with those of Zhao et al.^19^. The research method in this paper is the same as that in Reference^19^, but the calculation method of external power of potential sliding soil is slightly different. For unsupported slopes, different fissure depths and different levels of seismic force are compared, and the results are as shown in Fig. 6. The results show that the maximum difference between this paper and the research results in Reference^19^ is 0.96%, which shows that the data in this paper is reliable.

### Stability of slopes under earthquake action

**Figure 7:**
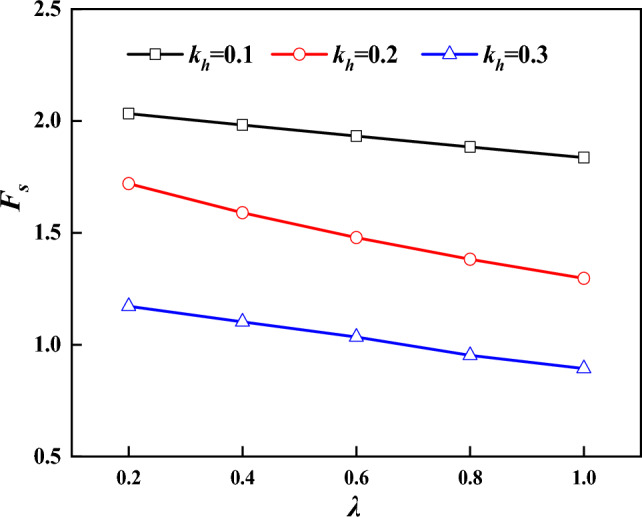
Relationship between $F_{s}$ and $\lambda$.

Using the algorithm proposed in this paper, the stability of slope is analyzed. Firstly, the stability of the slope under seismic action is analyzed. In previous studies, the vertical seismic action is often neglected on the analysis of the seismic action, which may lead to inaccurate calculation results. The $k_{h}$ are 0, 0.1, 0.2 and 0.3, respectively. Figure 7 presents the stability coefficients of intact supported slopes (without fissures, $\xi = 0$) for different values of $\lambda$. It can be seen that with the increase of $\lambda$, the stability coefficient $F_{s}$ of slopes basically shows a linear decreasing trend, and when $\lambda$ increases from 0.2 to 1.0, the stability coefficient decreases by 11%, which indicates that the seismic acceleration coefficient has a significant effect on the stability coefficient of slopes. The stability of the slope will be overestimated if the vertical seismic action is ignored on the analysis of the seismic action, and $\lambda { = }0.5$ will be taken for calculation later.

**Figure 8:**
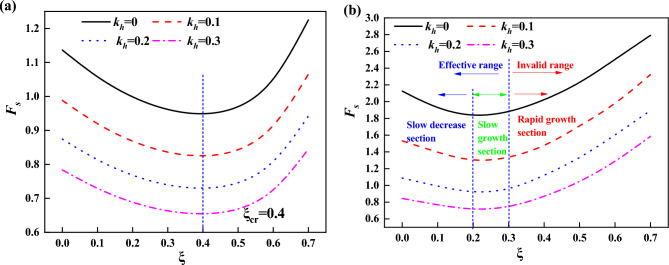
Relationship between $F_{s}$ and $\xi$ (**a**) unsupported (**b**) supported.

When the fissure nature of the loess is considered and the fissure generation is considered as part of the slope damage mechanism, for unsupported slopes, the relationship between slope stability coefficient and fissure depth for different seismic acceleration factors is shown in Fig. 8a, (where $\xi$ is used to denote the relative depth of fissure). From Fig. 8a, it can be seen that there is a critical value for $\xi$, which is noted as $\xi_{cr}$, and here $\xi_{cr} = 0.4$. The minimum value of the stability coefficient $F_{s\min }$ is obtained when $\xi = \xi_{cr}$ . The stability coefficient decreases with the increase of $\xi$ when $\xi < \xi_{cr}$, and increases with the increase of $\xi$ when $\xi > \xi_{cr}$. Since fissure is an unfavorable factor, for $\xi > \xi_{cr}$ , a larger fissure depth does not actually improve the stability of the slope. Utili^12^ considers that it is an effective fissure depth ($\xi < \xi_{cr}$) that makes the slope destructive. For $\xi > \xi_{cr}$, the failure mechanism obtained is not critical, and the results only represent the mathematically minimized solution of $F_{s}$.

Figure 8b gives the case of framed prestressed anchor supported slopes, and it can be seen that as $\xi$ increases, the $F_{s}$ curve is roughly divided into three segments: a slowly decreasing segment ($0 \le \xi < 0.2$), a slowly increasing segment ($0.2 \le \xi < 0.3$), and a rapidly increasing segment ($\xi \ge 0.3$). For the case of $\xi \ge 0.3$, it is considered that a larger fissure depth does not improve slope stability for the same reason as above. Therefore, the fast-growing section can be regarded as the ineffective fissure section, and the slowly decreasing and slowly growing sections can be regarded as the effective fissure section, in which the slope stability coefficient changes slowly and slightly.

For unsupported slopes, the reduction of slope stability by fissures as an unfavorable factor is obvious, while for slopes supported by framed prestressed anchors, the slope soil is reinforced by implanting anchors (pre-tensioned if necessary) in the soil mass. The support structure, as a safety reserve of the slope, assumes the role of resisting the sliding of the slope, and this role is stimulated when the slope generates a small displacement^24,25^. The growth of the fissure within a certain range allows the role of the support structure to be played. In other words, the slope supported by frame prestressed anchor can ensure the stability of the slope within a certain range during the development of fissures.

### Stability analysis of slope considering fissures

**Figure 9:**
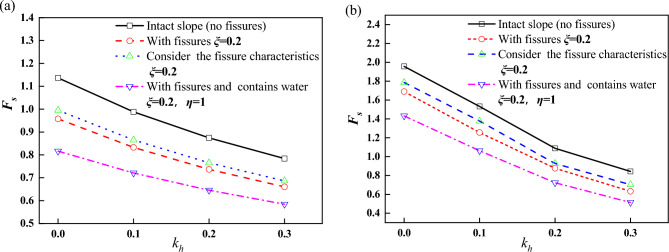
Relationship between $F_{s}$ and $k_{h}$ (**a**) unsupported (**b**) supported.

The stability of the three cases described in Section "Calculation assumption" for different seismic acceleration factors is illustrated below. The first case is dry fissures. The second case is to consider the fissure characteristics of loess, take $\xi = 0.2$. The third case is water-filled fissures, it is assumed that the fissures are filled with water. i.e., $\eta = 1$. As shown in Fig. 9, as expected, whether it is a supported slope or an unsupported slope, compared with a complete slope, the solutions of the existing fissures and considering the loess fissure have a lower stability coefficient.

From Fig. 9a, for unsupported slopes, $F_{s}$ gradually decreases with the increase of $k_{h}$. For example, for $k_{h} = 0.2$, compared with the intact slope, the safety factor of the slope considering loess fissures is reduced by 12% under the same earthquake acceleration coefficient. The stability coefficient decreases by 15% when the slope has fissures and by 25% when the fissures are filled with water, which indicates that the presence of fissures significantly decreases the slope stability. From Fig. 9b, it can be seen that the support structure reduces the adverse effect of fissure on slope stability to some extent, and the slope stability coefficient shows a small value of decrease.

**Figure 10:**
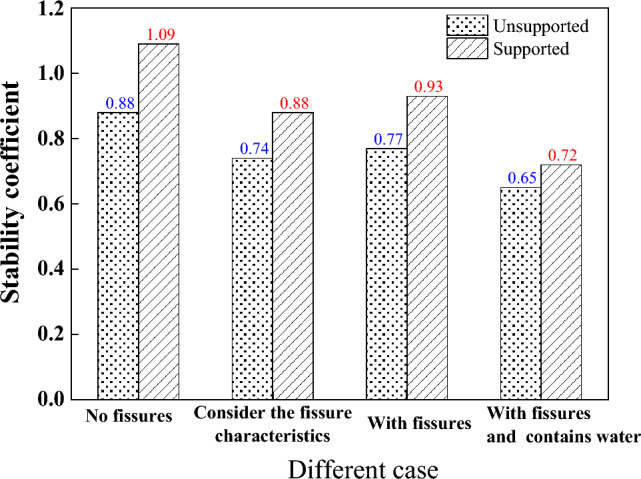
Safety factor of slope with or without support.

When the fissure contains water, the seepage effect of water will greatly reduce the stability of the slope. Taking $k_{h} = 0.2$ as an example, Fig. 10 gives the safety coefficients of slopes with or without support in different situations. The presence of support structures significantly improves the stability of slopes. For example, compared with the slope without support, when the slope has no fissures, the safety factor is increased by 24%. When the slope has fissures (without water), the safety factor is increased by 21%.

**Figure 11:**
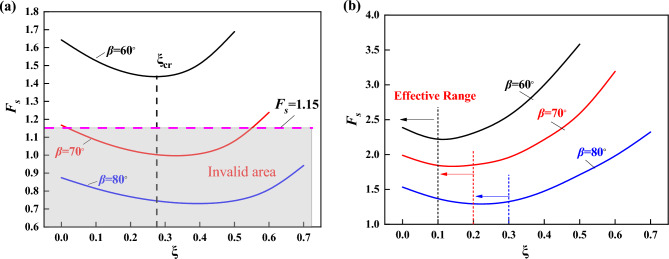
Relationship between $F_{s}$ and $\xi$ when $\beta$ is different (**a**) unsupported (**b**) supported.

The following analysis is carried out for the case of considering loess fractality, taking the fissure generation as part of the slope damage mechanism. When the slope angle $\beta$ is varied, with $k_{h} = 0.2$ and $\lambda = 0.5$, the relationship between $F_{s}$ and $\xi$ is shown in Fig. 11. It can be seen that when $\beta$ increases, the value of $\xi_{cr}$ increases and the slope safety factor decreases, and when Fs is less than 1.15, it is an invalid area. From Fig. 11b, it can be seen that the safety factor of the slope supported by frame prestressed anchors keeps moving back the effective fissure zone when $\beta$ increases. For example, the effective fissure length keeps increasing. Under this damage mechanism, compared with the steep slope, the fissure of the gentle slope is closer to point *A* in Fig. 3. The smaller the critical fissure depth is, the steeper the slope is, and the lower the stability coefficient is.

**Figure 12:**
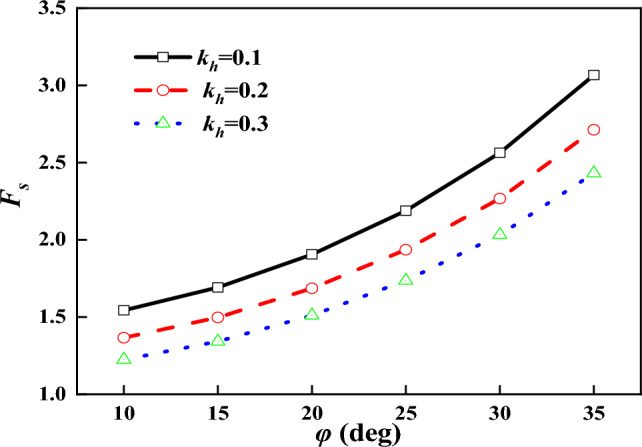
Relationship between $F_{s}$ and $\varphi$.

Because the same supporting structure parameters are used in the calculation, the steeper the slope is, the more obvious the influence of the supporting structure is, and the larger the effective crushing zone is. While for the gentle slope, its critical fissure depth is smaller and the impact made by the support structure is smaller, with conservative design parameter of the support structure at this time. Figure 12 gives the change curve of stability coefficient of the supported slope when the angle of internal friction $\varphi$ is changed, and it can be seen that the stability coefficient gradually increases with the increase of the angle of internal friction.

### Influence of the design parameters of the support structure

**Figure 13:**
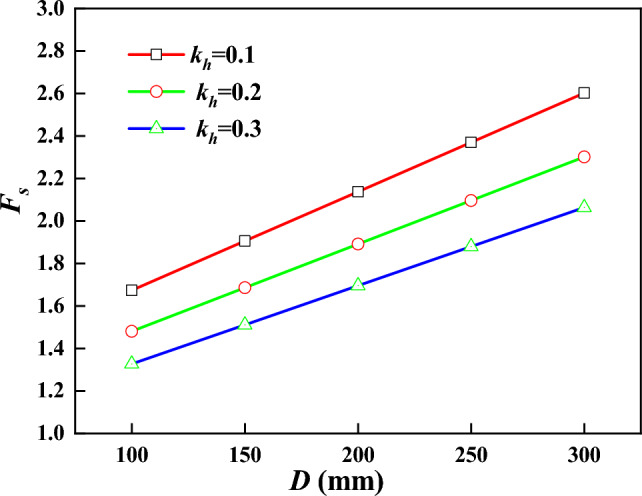
Relationship between $F_{s}$ and *D.*

**Figure 14:**
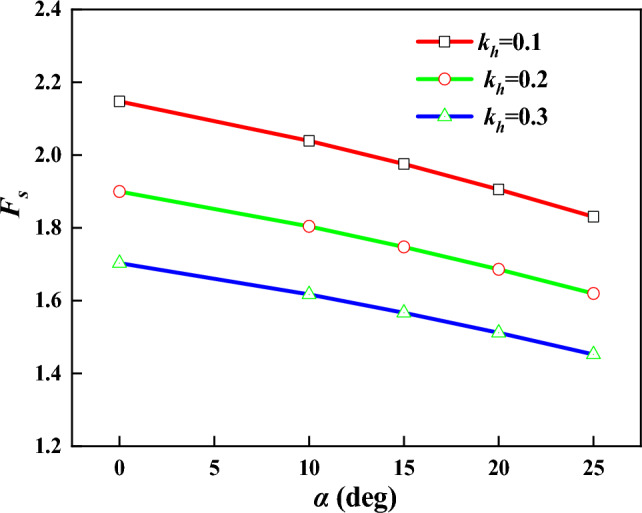
Relationship between $F_{s}$ and $\alpha$.

For loess high-fill slopes supported by frame prestressed anchors, the design parameters of the support structure will have an effect on the stability coefficient of the slope. Figures 13 and 14 show the variation curves of slope stability coefficients for different anchorage section diameter *D* and anchor rod inclination angles $\alpha$ considering the fractality of loess fill slopes, respectively.

From Fig. 13, it can be seen that with the increase of anchorage section diameter, the slope stability coefficient shows a linear growth trend, and the increase of anchorage section diameter makes the contact area between the surface of anchor and soil increase, which in turn increases the anchor pullout resistance. The energy consumption of anchor resistance increases, and the stability coefficient becomes larger.

From Fig. 14, it can be seen that the larger the inclination angle of anchor is, the smaller the stability coefficient is. When the inclination angle of the anchor gets larger, the component force of the anchor pullout resistance in the horizontal direction becomes smaller, with weaker support effect on the slope.

## Conclusions

By establishing a model for the stability analysis of the loess fill fissured slope supported by frame prestressed anchors, the following conclusions are obtained:

(1) In the stability analysis of loess fill slope, the fissure nature of loess is an important factor affecting the slope stability. Whether it is a supported slope or an unsupported slope, when considering the fracture properties of loess, the safety factor solution closer to the real failure mode of slope can be obtained.

(2) Ignoring the fissure nature of loess will overestimate the stability of loess fill slope. For unsupported loess filled fissured slopes, the relative fissure depth $\xi$ has a critical value $\xi_{cr}$. When $\xi < \xi_{cr}$, it is an effective fissure section, and the stability coefficient decreases with the increase of $\xi$. When $\xi > \xi_{cr}$, it is an invalid fissure section.

(3) Compared with the unsupported slope, the stability of the loess fill fissured slope supported by frame prestressed anchors can be improved by about 20%, which proves that the supporting structure can effectively control the stability of the loess fill fissured slope.

(4) The research results of this paper can enrich the stability analysis theory of loess filled fractured slope supported by frame prestressed anchor in loess area, and its research methods can be applied to different geological conditions and engineering backgrounds.

## Data Availability

All data generated or analysed during this study are included in this published article.
